# Nasal food challenge with hen's egg white allergen

**DOI:** 10.1186/s13223-024-00945-0

**Published:** 2025-01-12

**Authors:** Edyta Krzych-Fałta, Andrzej Namysłowski, Sławomir Białek, Monika E. Czerwińska, Konrad Furmańczyk, Aleksandra Tylewicz, Adam Sybilski, Bolesław Samoliński, Oksana Wojas

**Affiliations:** 1 Department of Basic Nursing, Medical University of Warsaw, Zwirki I Wigury 61, 02-097 Warsaw, Poland; 2 Department of Prevention of Environmental Hazards, Allergology and Immunology, Medical University of Warsaw, Zwirki I Wigury 61, 02-097 Warsaw, Poland; 3 Department of Biochemistry and Pharmacogenomics, Medical University of Warsaw, Zwirki I Wigury 61, 02-097 Warsaw, Poland; 4 Institute of Information Technology, Warsaw University of Life Sciences, Nowoursynowska 166 str, 02-787 Warsaw, Poland; 5 Second Department of Pediatrics, Centre of Postgraduate Medical Education, Wołoska 137 Str, 02-507 Warsaw, Poland

**Keywords:** Food allergy, Nasal allergen challenge, Nasal food challenge

## Abstract

**Background:**

Nasal allergen provocation tests are an important part of the diagnostics of allergic diseases triggered by environmental factors. Recently, increased attention has been paid to the potential use of this method in the diagnosis of food allergy. The objective of the study was to evaluate the usefulness of the nasal allergen provocation test in a group of subjects allergic to hen's egg white allergens.

**Methods:**

The material consisted of a group of 57 subjects (32 subjects with hen's egg white allergy and 25 healthy controls). The method consisted in a nasal allergen provocation test carried out with the use of hen's egg white allergen and assessed using the visual analog scale and optical rhinometry as well as by determination of sIgE and tryptase levels in nasal lavage fluid.

**Results:**

Subjective nasal symptoms and objective evaluations following the application of 100 µg of hen's egg white allergen revealed a moderately positive nasal mucosal response in optical rhinometry tests (ΔE = 0.34 OD).

**Conclusions:**

Nasal food challenge with hen's egg white allergen is a good diagnostic alternative in the group of food allergy patients. Due to the insufficient number of studies carried out so far, further attempts at standardization of the method are required.

**Supplementary Information:**

The online version contains supplementary material available at 10.1186/s13223-024-00945-0.

## Introduction

Due to its increasing prevalence and pathomechanism involving a complex combination of genetic, epigenetic, and environmental factors, food allergy (FA) constitutes to be a significant clinical problem [1]. Self-reported food allergy is far more common than physician-diagnosed FA or diagnosis at formal food challenge; symptoms of FA are reported on the average by 17.2% (16.0–17.6) of all examined subjects; sIgE-confirmed allergy is diagnosed in 4.1% (3.2–5.1) of patients while simultaneous clinical manifestation and sIgE expression are detected in 2.2% (0.8–3.7) of patients. FA as confirmed by an oral food challenge is diagnosed in 0.9% (0.8–1.0) of all subjects [2]. In other studies, FA symptoms are reported population-wise in an average of 13% of adults and 12% of children, with sensitization to peanut allergens being reported for 0.6%, sensitization to milk being reported for 3%, sensitization to hen's egg being reported for 1%, sensitization to fish being reported for 0.6%, and sensitization to shellfish being reported for 1.2% of patients. In contrast, oral food challenge confirms sensitization to milk and fish allergens respectively at 0.9% and 0.3% of the total population [3–5]. The problem of allergy to hen's egg white relates mainly to children in infancy and early childhood, in whom the prevalence of sensitization to this allergen is estimated at 16.5% [95% CI 15.1–17.9], with diagnosis being confirmed by an oral food challenge using the respective allergen in 8.9% [95% CI, 7.8–10.0] [3, 6]. The problem of allergy to hen's egg whites is also encountered within the adult population. Symptoms of FA in the general population as confirmed by skin prick tests or sIgE expression assays are diagnosed in nearly 3% of the general population, with hen's egg white allergy, for example, being detected in 0.9% of subjects [3–5].

Skin prick tests, sIgE expression, elimination diets, and oral food challenges play an important role in the diagnostics of FA and identification of the causative allergens. The latter, despite the risk of a number of adverse effects including anaphylactic shock, remains the gold standard in FA diagnostics [7, 8]. In an attempt to identify alternatives to oral food allergen provocation tests, increasing attention is being paid to laboratory methods, including basophil activation tests using flow cytometry techniques [6] and nasal allergen challenge (NAC) [9–14].

The objective of the study was to evaluate the usefulness of the NAC in a group of subjects allergic to hen's egg white allergens. Implementation of the NAC scheme as widely used in the diagnostics of allergic rhinitis in diagnosing food allergies (nasal food challenge, NFC) is an alternative to oral food challenge; as shown by preliminary studies, the NFC is characterized by a high safety profile and no risk of potential systemic reactions. However, it should be noted that a history of anaphylactic shock is an absolute contraindication in the generally accepted eligibility criteria for intranasal testing [13–15]. Therefore, one should infer that only patients with no history of anaphylactic shock were included in the group of subjects eligible for NFC. The experiment carried out in laboratory conditions using a dose of extracted allergen and assessed using the visual analog scale (VAS), optical rhinometry (OR), and determination of sIgE and tryptase levels in nasal lavage fluid provided a good setting for the assessment of the early phase within all aspects of allergic reaction.

## Materials and methods

### Ethical statements

The study was conducted in accordance with the Declaration of Helsinki. Approval of the Bioethics Committee of the Medical University of Warsaw (KB 63/2022: approval date: 16 May 2022) was obtained.

### Participant recruitment

Included in the study were a group of 57 subjects, with 32 patients allergic to hen's egg white allergens and 25 healthy controls (a group of allergy-free subjects in whom sensitization to any allergens was excluded). The inclusion criteria were as follows: clinical history confirming the allergy to hen's egg white allergens, positive results of hen's egg allergen skin prick tests(Allergopharma; mean wheal and flare diameters 3.8 mm and 7 mm, respectively, sIgE (Polycheck) 3.5 kU/L—class 2, and stable atopic dermatitis in remission). In order to reduce the risk of non-specific response of nasal mucosa to hen's egg white allergens, patients who had been diagnosed with allergic rhinitis or any other nasal and sinus diseases were excluded from the study. On the other hand, the exclusion criteria included a history of anaphylactic shock, external nasal deformities and structural abnormalities of the nasal cavity (including the deviation of the nasal septum and hypertrophy of the inferior nasal turbinate), atrophic rhinitis, history of immunization within the last week, history of surgery within less than 8 weeks, active acute upper respiratory tract infection or history of acute upper respiratory tract infection within 4 weeks prior to the study, uncontrolled asthma, severe systemic conditions, and systemic immunotherapy [13, 14]. Pre-study recommendations included the withdrawal of pharmaceutical agents (antihistamines 7 days prior, mast cell stabilizers 2 days prior, intranasal glucocorticosteroids 2 days prior, NSAIDs 2 days prior, CsA 7 days prior, systemic antihistamines 7 days prior, systemic glucocorticosteroids 14 days prior, antileukotrienes 21 days prior) [16] and a hen's egg elimination diet. Active anterior rhinomanometry was used to assess nasal patency (Lungtest 1000 MES, pressure changes between ± 25 – ± 250 Pa at 150 Pa and a Broms’ circle radius of 0.2 L/s, with the intersection between the circle and the pressure/flow function being used as the point for calculation of nasal resistance, Table 1, 2), with no significant nasal patency abnormalities being demonstrated in the study group.

**Table 1:** Clinical indicators of functional tests of the respiratory system

	Patients allergic to hen's egg allergens (N = 32)	Control group (N = 25)
FENO nose	339.12	297.76
SD	80.121	65.009
FENO lung	21.84	20.88
SD	2.248	2.127
spirometry (FEV1/FVC)	98.24 / 43th percentile	99.88 / 48th percentile
SD	7.617 / 31.315	8.181 / 32.343
spirometry (FEV1)	99.8	93.08
SD	14.947	17.296

Rhinomanometry								
Parameter	Unit	V	SD	Current	V	SD	Current	
RnRSIn*	kPa/L/s	6.481	0.045	0.660	4.193	0.026	0.526	
RnRBIn*	kPa/L/s	9.500	0.058	0.600	13.347	0.045	0.357	
RnRSEx*	kPa/L/s	8.187	0.053	0.682	6.400	0.032	0.474	
RnRBEx*	kPa/L/s	10.186	0.055	0.545	15.477	0.064	0.403	
RnLSIn*	kPa/L/s	5.320	0.093	1.543	9.465	0.059	0.774	
RnLBIn *	kPa/L/s	9.340	0.145	1.527	17.77	0.069	0.719	
RnLSEx*	kPa/L/s	12.615	0.087	0.584	9.905	0.101	0.664	
RnLBEx*	kPa/L/s	15.846	0.099	0.611	10.067	0.043	0.522

**Table 2:** Study group characteristics

Parameter	Patients allergic to hen's egg white allergen (N = 32)	Control group (N = 25)
Age Mean SD	25.640	26.6
	9.982	9.840
Height SD	167.72	169.32
	12.644	12.344
Body Weight SD	68.80	67.6
	18.42	16.733
BMI SD	24.21	23.296
	5.390	3.853

### Procedures

The method used in the study consisted in a NFC with hen's egg white allergens. The early phase of the allergic reaction was evaluated in a comprehensive manner [5] using the subjective VAS scale (0—no discomfort, 10—maximum intensity of the three key nasal symptoms: itching, discharge and obstruction) as well as an objective measure (OR, Rhinolux Rhios GmbH, Grosserkmannsdorf, Germany). OR accurately determines the onset (T1) and end (T2) of the allergic reaction as well as the degree of the swelling of the nasal mucosa as measured by optical density (OD, ΔE). The principle of the exam is that of emission spectrometry, in which a beam of infrared rays emitted from a transmitter towards a detector, both placed on opposite sides of the nose, passes through nasal structures and records any changes in flow within the blood vessels. The change in the flow assumes positive values (in the course of NAC), negative values (following anemization of nasal mucosa) or alternating values as observed in the nasal cycle. As previously mentioned, changes in the infrared beam absorption curve (wavelength range of 600–1000 nm) in NAC are the consequence of swelling of nasal cavity mucosa [17, 18]. The device provides a convenient way to assess changes in nasal patency in real time, and objectivizes the course of the NAC regardless of the degree of swelling and the patient's cooperation level. In addition, material in the form of nasal lavage fluid was collected using the Greiff technique [19] for further testing by means of immunoenzymatic ELISA assays (UNICAP, Sweden) for determination of sIgE (calibration curve: CAL-0.00, 0.01 CONC, 7 mean, CALC 0.001(> 99%CV); CAL-0.35, 0.01 CONC, 153 mean, CALC 0.35 (0.6%CV); CAL-0.70, 0.70 CONC, 302 mean, CALC 0.71(1.8%CV); CAL-3.50, 3.50 CONC, 1421 mean, CALC 3.46(2.1%CV); CAL-17.5, 17.5 CONC, 5806 mean, CALC 17.5(2.0%CV); CAL-100, 100 CONC, 16394 mean, CALC 100.0(0.6%CV) and tryptase (calibration curve: CC-1, 2311 RESP, 13.7 CONC, 1.2%CV). Saline solution, administered bilaterally into the nasal cavity in amounts of 5 mL, was used in the lavage procedure.The obtained material was centrifuged in laboratory conditions (temperature of 4ºC in the centrifugation chamber at maximum speed, time range 15 min) and then the obtained supernatant was frozen at −20 ºC. The thus obtained material was subjected to further examinations in laboratory conditions (immunoenzymatic ELISA assays).

### Assays

The study design followed the NAC standards [5, 14] and included observation of the early phase of the allergic reaction. Following local acclimatization (20 min, room temperature of 21 °C, relative humidity of 46%), nasal patency was assessed and nasal lavage was performed. The next stage of the study involved the evaluation of subjective discomfort as measured using the VAS scale and of the changes in nasal patency as assessed using OR at consecutive time points, i.e. immediately after administration of the study solution as well as 15, 30, 45, 60, and 75 min into the study. The NFC was delivered in the form of a titration challenge with changes in nasal mucosal reactivity and the degree of nasal patency being evaluated in response to topical application of hen's egg white allergen at the doses of 12.5 µg, 25 µg, 50 µg, and 100 µg using a standardized atomizer (0.1 mL administered bilaterally into the nasal cavity). Successive, increasing doses of the allergen were administered until the threshold response of nasal mucosa to the applied hen's egg white allergen was observed. The allergen for the test was prepared from raw hen's egg by lyophilization in laboratory conditions and stored for a maximum of 24 h due to the lack of a preservative for the challenging agent.The material (lyophilisate) for the study was obtained from an organically grown chicken egg, which was subjected to filtration processes in laboratory conditions and the final result was: egg_white_F1.2: 2.60 g – egg white homogenate with PBS (1:4) filtered through 5 µm and 1.2 µm filters and egg white 4.00 g (pure egg white, not homogenized with PBS, without filtration, lyophilized); yolk_F1.2: 6.33 g yolk homogenate with PBS (1:4) filtered through 5 µm and 1.2 µm filters and yolk 6.90 g (pure yolk not homogenized with PBS, without filtration, lyophilized). This material was the basis for the preparation of dilutions (in saline) using a laboratory scale for NFC. Physiological saline was used as the solvent for lyophilizate solution. Capillary electrophoresis of the lyophilizate was used to confirm the presence of hen's egg [20]. The outcomes of the challenge were evaluated in line with the criteria set forth in the *EAACI Position paper on the standardization of nasal allergen challenges*, with NAC being classified as strongly positive for subjective symptoms being assessed at 55 mm and the decrease in nasal patency or increase in air flow velocity as measured by objective techniques at 40%. Outcomes were interpreted as moderately positive when nasal symptoms were assessed at 23 mm and the decrease in patency/increase in air flow velocity was 20% [14]. Changes in nasal patency as measured by OR are referred to as strongly positive when the change in infrared beam absorption is at the level of 0.52 OD [17]. This research was funded by the National Science Center Miniature-5 grant number 2021/05/X/NZ5/009.

### Statistics

In the statistical analysis, positional statistics (quartiles) were determined and box plots were created for the VAS scale results for three separate symptoms, the differences (delta E) in OR measurements at the experimental time points of 1 min, 15 min, 30, min, 45 min, 60 min, and 75 min and the sIgE/tryptase levels in the early-phase allergic reaction. Comparisons were carried out using the paired Wilcoxon test with the significance level of 0.05 (i.e. differences were reported as significant when the corresponding p-value was lower than 0.05).

The area under the ROC curve (AUC) was used to assess the differences between allergy patients and controls in terms of symptoms on the VAS scale, ΔE values in OR and sIgE/tryptase levels as assessed at specific time points. Descriptive statistics (means and standard deviations [SD]) were used to summarize the characteristics of patients. The two-sample Welch's t-test was used for comparisons between allergy patients and the control group. All analyses were performed using the R software package.

## Results

No significant symptoms suggesting anaphylactic shock were observed during NFC. All patients were monitored in hospital and did not exhibit any alarming symptoms during the late phase of the allergic reaction.

### Participant characteristics

In the assessment of nitric oxide levels as expired from the upper respiratory tract (Provita, measurements of maximum exhaled nitric oxide, averaged expiratory flow velocity (VE), averaged flow velocity of nitric oxide (VNO), sensor measurement range up to 6000 ppB), no significant deviations from reference values were identified, with values amounting to 319.538 (SD 47.935) ppB for women and 374.818 (SD 94.0290) ppB for men. No ventilation abnormalities were also demonstrated in the functional examination of the lower airways: spirometric examination (Lungtest 1000 MES, measured flow range ± 18 L/s, dflow measurement accuracy < 2%, flow measurement resolution ± 10 mL/s, measured volume range ± 10 l, volume measurement accuracy < 2%) excluded bronchial obstruction (FEV1/FVC: 99 [SD 7,314] in women, 96,909 [SD 8,372] in men; FEV1: 98 [SD 14,106] in women and 100,727 [SD 16,577] in men). Similarly, the nitric oxide levels in air exhaled from the lower respiratory tract as an inflammation marker were within normal limits, amounting to 21.307 (SD 2.135) ppB for women and 22.181 (SD 2.272) ppB for men. No statistically significant differences were observed between the group of subjects sensitized to hen's egg white allergens vs. the control group (Table 1).

In the group of sensitized women (52% of the subjects), the average age was 25.615 years (SD 7.588) as compared to 26.181 years (SD 12.905) in men. The body mass index values amounted to 24.092 (SD 5.33) and 24.4 (SD 5.964) in females and males, respectively. In the control group, a significant difference was observed in relation to body mass index between female and male subjects (p = 0.013). The study group did not differ in terms of examined characteristics at a statistically significant level (Table 2).

### Subjective and objective assessment of the early phase of the allergic reaction

With the increasing doses of hen's egg white allergen used in the NFC, a significant increase was observed in the severity of nasal symptoms and variation in flow rates as measured by OR together with a no differences in sIgE (remaining at the level of < 1.12 kUA/L, per level 0.07—0.12 kUA/L) and slight tryptase levels in nasal lavage fluid (a slight increase was observed in only four subjects per level 2.64—3.03 μg/L, Table S3), which demonstrates a moderate response from nasal mucosa only in selected cases within the study group of subjects allergic to hen's egg white proteins.

**Fig. 1:**
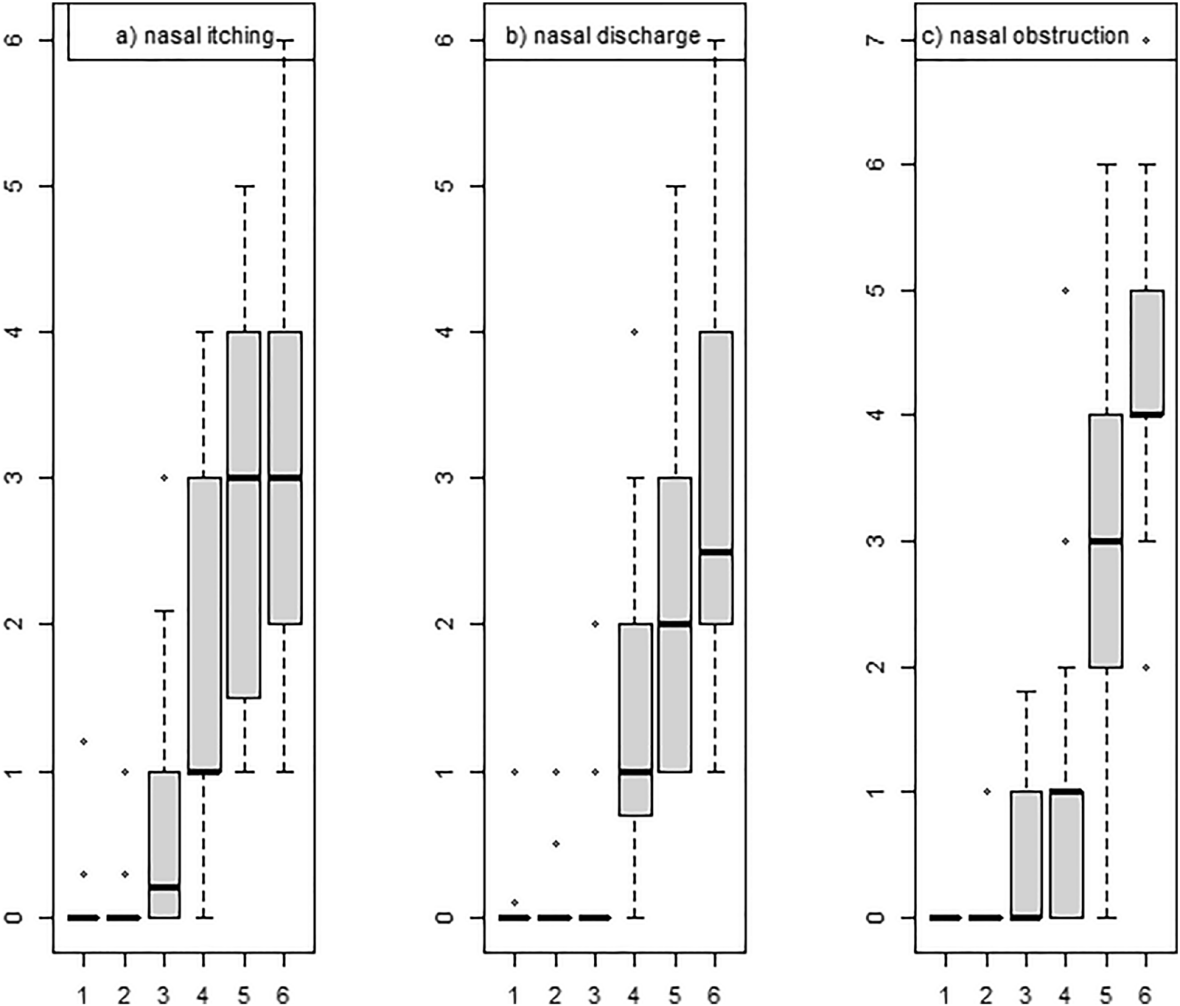
Assessment of subjective complaints as measured using the visual analog scale

Medians for each of the measured characteristics were used for subjective and objective evaluations (ΔE, nasal itching, discharge, obstruction in VAS scale). The first recorded nasal symptom was nasal itching (at a dose of 12.5 µg), followed by watery discharge and nasal congestion (dose of 25 µg). For nasal itching, the sequential median values amounted to 0.2, 1.0, and 3.0, with the highest level being measured 75 min into the test (significant difference vs. the control group) following the application of a dose of 100 µg (nasal mucosal reactivity thresholds were relatively diverse within the study group of subjects allergic to hen's egg white proteins Fig. 1.).

On the other hand, the median values related to nasal discharge and nasal obstruction significantly differentiated the study groups within the period of 45 to 75 min into the early allergic reaction phase amounting to 1.0, 2.0, and 2.5 vs. 1, 3, and 4 at 45, 60, and 75 min. The dominant symptom assessed using the VAS was nasal obstruction, which persisted to the greatest extent in the group of patients allergic to hen’s egg protein (mean 5 cm, range: 2–7 cm on the VAS), at the administered dose of 100 µg vs. the control group.

**Fig. 2:**
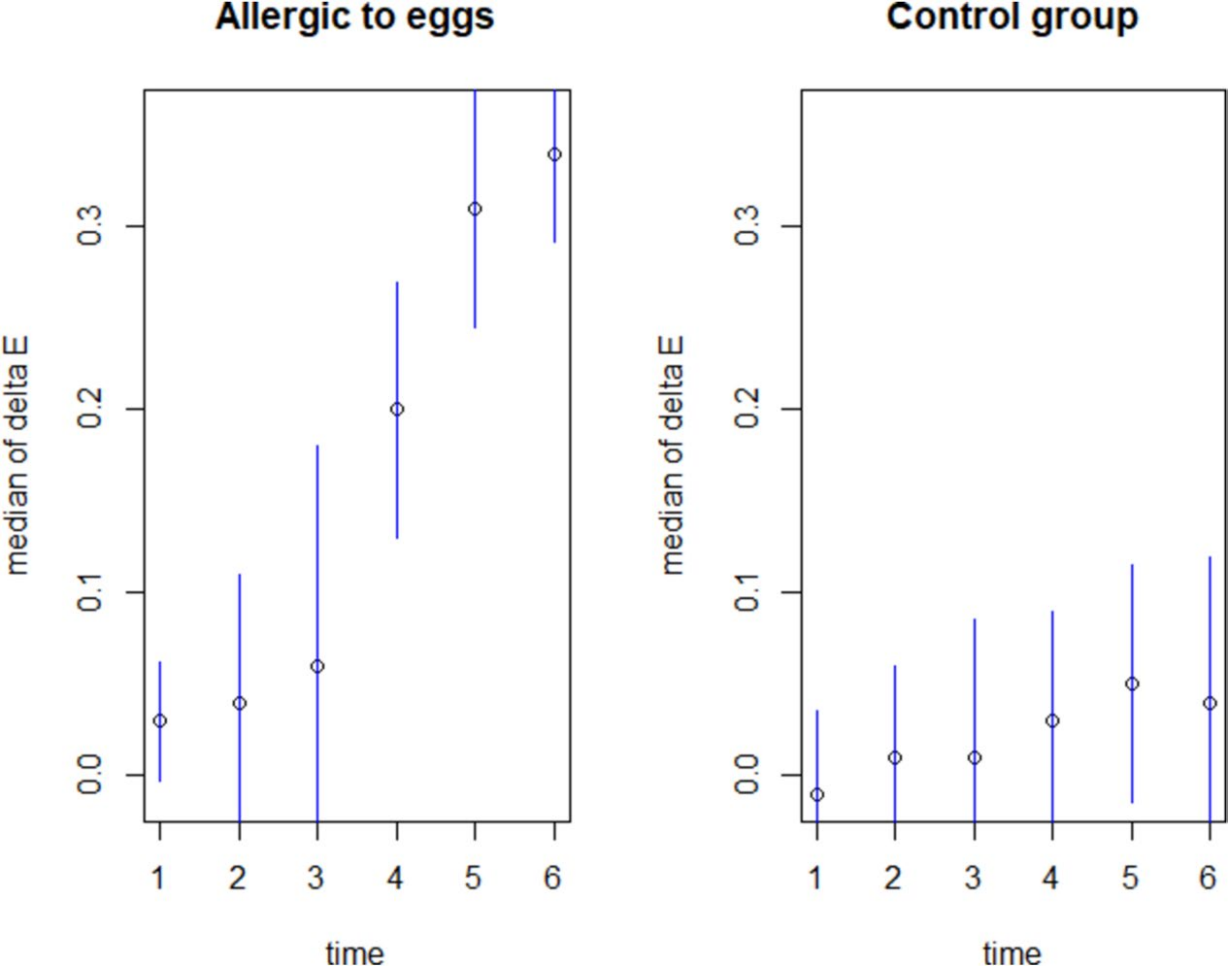
Objective assessment of changes in nasal patency as measured by optical rhinometry

Significant changes in nasal patency as measured by OR were recorded prior to the subjective complaints as measured by the VAS scale being reported (i.e. starting from 30 min into the study) vs. control group. Differences in the variability in blood flow within the blood vessels were observed, with values amounting to 0.03; 0.04; 0.06; 0.20; 0.31; 0.34 OD in the study group vs. − 0.01; 0.01; 0.01; 0.03; 0.05; 0.04 OD in the control group (Fig. 2.).

We observed significant statistically changes in the study group for differences in median for 45 min to 15 min (p = 2.88e-05), for 60 min to 15 min (p = 3.291e-05) and for 75 min to 15 min (p = 3.294e-05). In the control group, minor and statistically insignificant changes in nasal patency were observed as being due to the variation in flows associated with the physiological nasal cycle (p > 0.05).

Changes observed following NFC were significantly different as compared to the control group: nasal itching (VAS scale) p = 0.0001846 at 45 min, p = 1.208e − 05 at 60 min, p = 6.177e − 05 at 75 min; nasal discharge p = 3.92e − 05 at 60 min, p = 0.0005181 at 75 min; nasal obstruction p = 3.92e − 05 at 60 min and p = 1.165e − 05 at 75 min; nasal flow variability (OR) p = 2.88e − 05 at 45 min, p = 3.291e − 05 at 60 min, and p = 3.294e–05 at 45 min.

### Comparison of outcomes to determine nasal challenge

**Fig. 3:**
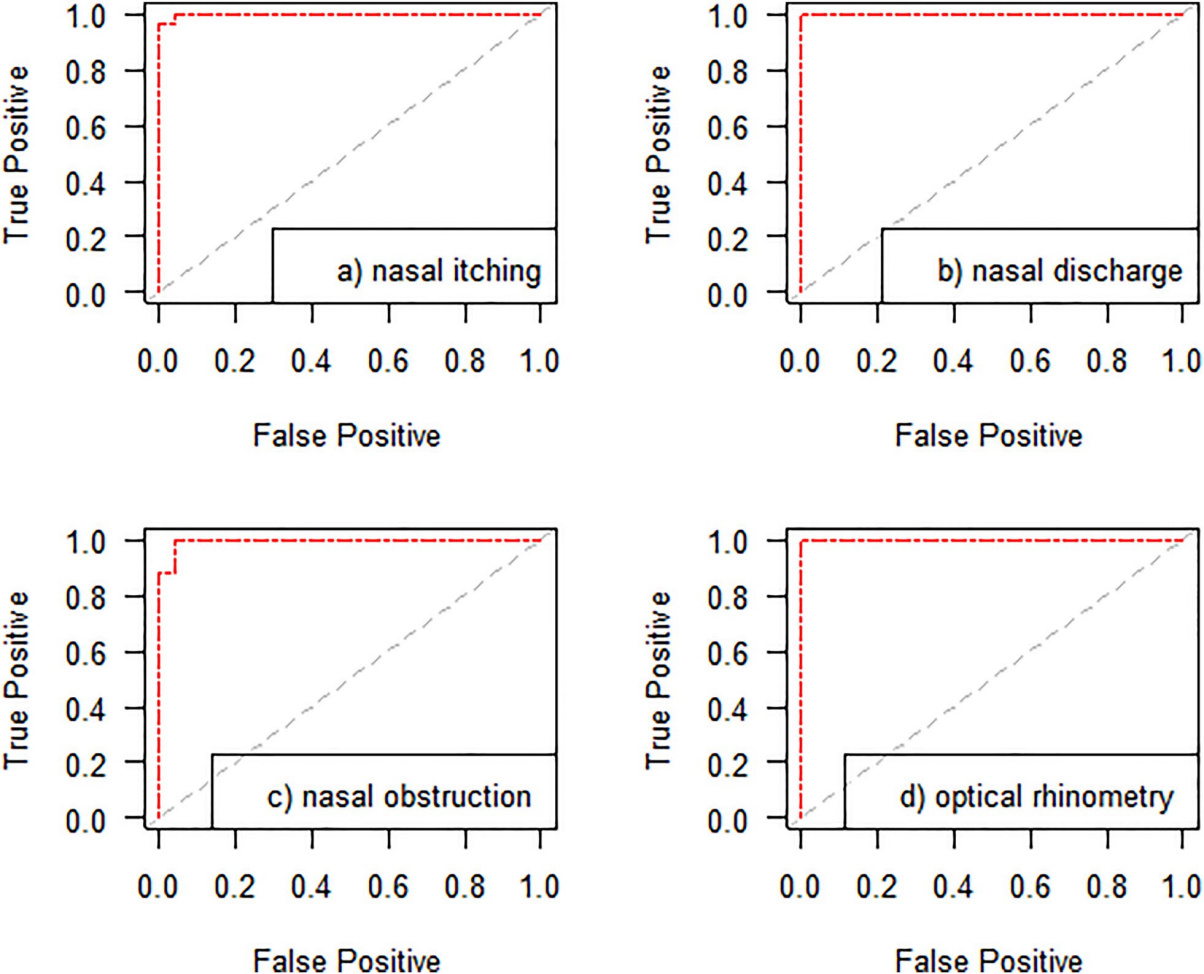
specificity and sensitivity of nasal food challenge. Due to the lack of a significant increase in sIgE and tryptase in nasal lavage fluid, no ROC curve was determined

In the comparison of the VAS and OR tools, the former was shown to perform definitely worse in terms of differentiating the study groups, with the AUC for the overall symptoms as assessed using the VAS scale at 45 min amounting to 0.70. In the analysis of symptoms as measured using the VAS scale, the study groups differed with respect to nasal itching at 30 min (AUC = 0.4752), 45 min (AUC = 0.7616), 60 min (AUC = 0.9808), and 75 min (AUC = 0.9984); for nasal discharge, the AUC values were 0.224 at 30 min, 0.712 at 45 min, 0.976 at 60 min and 0.9952 at 75 min into the study. The greatest difference between the study groups with respect to nasal obstruction was observed at 75 min into the study (AUC = 1). Consecutive measurements of the area under the ROC curve amounted to AUC = 0.3344 at 30 min, AUC = 0.5712 at 45 min, AUC = 0.9136 at 60 min, and AUC = 1 at 75 min (Fig. 3a, b, c, d).

## Discussion

In the course of food allergies, including hen's egg white protein allergy, typical gastrointestinal symptoms such as nausea, abdominal pain, and diarrhea are observed. These can be accompanied by complaints such as itchy skin, hives, conjunctivitis, oral mucositis, and rhinitis. Atypical symptoms, including headaches or difficulty concentrating, have also been recorded. In the course of severe forms of food allergy, anaphylactic shock may occur, posing an immediate threat to the patient's health and life [1, 8]. Sensitization to egg allergens mainly refers to sensitization to hen's egg white proteins (ovomucoid [Gal d1], which retains its allergenic activity despite heat treatment (with the exception of the baking process), ovoalbumin [Gal d2], which, along with ovotransferrin (Gal d3), is partially resistant to digestion and thermolabile, and lysozyme [Gal d4]). In contrast to the pediatric population, the allergy to hen's egg white in adults is considered to be persistent in nature while primary sensitization to hen's egg white is extremely rare [21].

### Differential diagnosis of food allergies, including sensitization to hen's egg allergens

From the clinical point of view, it is important to properly identify the allergen so as to avoid it in the future and to appropriately treat any allergic reactions following accidental exposure. Due to the multitude of triggers and rich symptomatology, the diagnostics of food allergies, including sensitization to hen's egg white allergens, is a difficult and arduous process. The key elements in the diagnostics of hen's egg allergy include clinical history, skin tests, lab tests (sIgE/IgG4, BAT; CD63 and CD203c expression tests, component diagnosis, particularly in recurrent anaphylactic reactions) and/or elimination diets [1]. Double-blind placebo-controlled food challenge tests remain the gold standard. For safety reasons, however, these should only be administered within specialized centers under close medical supervision. Challenge tests are classified as either open or blinded challenges (single- and double-blind placebo-controlled challenges). Double-blind placebo-controlled food challenge (DBPCFC) is the gold standard in the diagnostics of food allergies. Thanks to its high sensitivity and specificity, it plays a key role in demonstrating cause-and-effect relationships between ingested food and clinical symptoms. Unlike open and elimination trials, the DBPCFC facilitates exclusion of the potential involvement of psychogenic factors. The test consists in randomized administration of the increasing doses of the verum, or the food suspected of causing disease symptoms, and of identical-looking and tasting placebo. However, when deciding on carrying out a challenge test, it is important that limitations of this are taken into account as being due to the possibility of developing anaphylaxis or another severe reaction, the possibility of false-negative and false-positive results, and the costs associated with performing the test in a hospital setting [22].

### Practical implications of nasal allergen challenge

In our work, we present the results of a pioneering and unique study demonstrating the possibilities related to the use of NFC in the diagnostics of food allergies, with the possibility for objectivization of the course of the challenge offering a characteristic and important alternative to the food challenge test. To date, few research data were available on this subject in international literature [9–12].

According to the consensus statement of the EAACI Position paper on the standardization of nasal allergen challenges, indications for NAC in the diagnostics of allergies include demonstration of allergy to airborne allergens in the course of persistent, chronic, occupational, and local rhinitis, differential diagnosis of ocular symptoms, determination of causal relationship between the allergen and the symptoms, especially in the case of difficulties in interpretation of skin patch tests and sIgE levels, determination of indications for immunotherapy, determination of allergens directly responsible for the symptoms for the establishment of the composition of allergen vaccine, and monitoring of the efficacy of specific immunotherapy. Of particular note is a new indication related to the diagnostics and the possibilities for the use of NAC in food allergies [14]. Among the methods for diagnosing allergic diseases, NACs are characterized by high sensitivity and specificity rates. Positive challenge results are obtained in the group of patients with strongly positive skin prick test/sIgE determination results for conjunctival and NAC [23].

By implementing the NAC principle in NFC, convenient conditions can be established for the objectivization of the diagnostic method. The course of NFC is typical for NAC and involves observation of nasal symptoms such as nasal itching, nasal discharge, and nasal obstruction in the early and late phases of the allergic reaction. The implemented test design, including the scales used to assess subjective complaints and the objective techniques, facilitates the assessment of the degree of the response of nasal mucosa to local allergen application. The NFC is in its nature a *titration test* and, appropriately to the allergen being tested, the degree of response is assessed using objective techniques and subjective scales at 15-min intervals. The selection of objective methods for the assessment of nasal patency in NFC may vary, consisting mainly of acoustic rhinometry [12] and computed thermography [9, 10]. A study by Gelis et al. involving NFC with seafood allergens revealed significantly significant decreases in nasal cavity cross sections. Similarly, Clark et al. demonstrated temperature differences within the nasal cavity area during the early phase of the allergic reaction following intranasal provocation with peanut allergens [10, 12]. Both studies revealed outstandingly positive outcomes of the challenge tests, warranting the possible use of this test method in the diagnostics of FA. In our study, optical rhinometry, directly illustrating quantitative changes in nasal patency as expressed by means of optical density, was used to assess objective changes in nasal patency. Unlike other techniques, such as acoustic rhinometry or rhinomanometry, OR does not require complex mathematical operations since the device delivers the final result as the change in patency of nasal cavity expressed in ΔE; in our case, the difference was 0.34 OD. Thus, one may conclude that the mucosal response in this particular case was 34%. In addition, the course of the early phase of the NFC was typical for NAC and was characterized by a slight increase in sIgE levels in the lavage fluid, showing the involvement of an IgE-dependent response in NFC. Most reports documenting the clinical significance of sIgE/tryptase levels in nasal lavage fluid relate to local rhinitis and occupational airway allergy [24–27].

Only the titratable nature of NFC was described in current art [10, 12], and the acquisition of lyophilizate for testing is carried out under laboratory conditions thus being restricted for scientific purposes. Our study, with a moderately positive response being recorded to the topically applied egg white allergen, contributes to the establishment of an appropriate environment for further research in the future, particularly in the area of allergen form and dose standardization so as to objectivize the cut-off for positive challenge tests. Importantly, the changes observed in the course of NFC are limited to the nasal cavity with only occasional systemic manifestations, thus confirming the method's high safety profile in contrast to oral food samples. Absolute contraindications [12] and appropriate preparation for the test, including an elimination diet just as in the case of oral food challenge, are important criteria to be taken into account when qualifying patients for NFC [20].

### Limitation of study

A specific limitation of the present study is related to the ability to assess the safety of the test due to the existing contraindications, including, as previously mentioned, the exclusion of patients with a history of anaphylactic shock [13, 14, 16]. A significant limitation of the study is the need to exclude patients diagnosed with allergic rhinitis from the study group. Another major limitation of the study consisted in the lack of a reference point for the nasal mucosal response to the oral hen's egg white allergen challenge test as carried out on the study group.

## Conclusions

The NFC provides a good opportunity for the assessment of the response of the nasal mucosa in food allergies. Implementation of the principles of the NAC in the NFC test will facilitate further standardization of the method. Nevertheless, further research is required in this field due to the small number of scientific reports available to date.

## Supplementary Information

Additional file 1.

## Data Availability

The data that support the findings of this study have been deposited in the Medical University of Warsaw with the primary accession under National Science Center Miniature-5 grant number 2021/05/X/NZ5/009. The data are contained in the manuscript or in the Supplementary Information files.
