# Correction: Nasal food challenge with hen’s egg white allergen

**DOI:** 10.1186/s13223-025-00984-1

**Published:** 2025-08-25

**Authors:** Edyta Krzych-Fałta, Andrzej Namysłowski, Sławomir Białek, Monika E. Czerwińska, Konrad Furmańczyk, Aleksandra Tylewicz, Adam Sybilski, Bolesław Samoliński, Oksana Wojas

**Affiliations:** 1 Department of Nursing Propedeutics, Medical University of Warsaw, Zwirki i Wigury 61 str, 02-097 Warsaw, Poland; 2 Department of Prevention of Environmental Hazards, Allergology and Immunology, Medical University of Warsaw, Zwirki i Wigury 61 str, 02-097 Warsaw, Poland; 3 Department of Biochemistry and Pharmacogenomics, Medical University of Warsaw, Zwirki i Wigury 61 str, 02-097 Warsaw, Poland; 4 Institute of Information Technology, Warsaw University of Life Sciences, Nowoursynowska 166 str, 02-787 Warsaw, Poland; 5 Second Department of Pediatrics, Centre of Postgraduate Medical Education, Wołoska 137 str, 02-507 Warsaw, Poland

**Correction to: Allergy, Asthma & Clinical Immunology (2025) 21:3**

10.1186/s13223-024-00945-0.

In this article [1], the grant number 2021/05/X/NZ5/01099 for National Science Center Miniature-5 grant number was incorrectly processed as 2021/05/X/NZ5/099 in “Materials and Methods” and “Availability of data and materials” sections.
