# Supplementary material for: Nasal food challenge with hen's egg white allergen
Source: Allergy Asthma Clin Immunol. 2025 Jan 12;21:3. doi: 10.1186/s13223-024-00945-0 (PMC11727154; doi:10.1186/s13223-024-00945-0)
Supplement: Supplementary file 1 [file 13223_2024_945_MOESM1_ESM.odt]

Table S3. sIgE and tryptase levels in nasal lavage fluid

| f75 (sIgE hen`s egg) (kUA/L) | tryptase levels (μg/L) |
| --- | --- |
| sIgE | class |
| 44 | 0.09 | <1 |
| 47 | 0.09 | <1 |
| 45 | 0.09 | <1 |
| 42 | 0.08 | 2.78 |
| 39 | 0.08 | <1 |
| 44 | 0.09 | <1 |
| 44 | 0.09 | 3.03 |
| 49 | 0.1 | <1 |
| 55 | 0.11 | 2.64 |
| 56 | 0.12 | 2.99 |
| 54 | 0.11 | <1 |
| 52 | 0.11 | <1 |
| 56 | 0.12 | <1 |
| 53 | 0.11 | <1 |
| 56 | 0.12 | <1 |
| 58 | 0.12 | <1 |
| 54 | 0.11 | <1 |
| 52 | 0.11 | <1 |
| 41 | 0.08 | <1 |
| 41 | 0.08 | <1 |
| 34 | 0.07 | <1 |
| 35 | 0.07 | <1 |
| 38 | 0.07 | <1 |
| 36 | 0.07 | <1 |
| 35 | 0.07 | <1 |
| 35 | 0.07 | <1 |
| 36 | 0.07 | <1 |
| 40 | 0.08 | <1 |
| 37 | 0.07 | <1 |
| 42 | 0.08 | <1 |
| 37 | 0.07 | <1 |
